# Dataset of cocoa aspartic protease cleavage sites

**DOI:** 10.1016/j.dib.2016.06.021

**Published:** 2016-06-24

**Authors:** Katharina Janek, Agathe Niewienda, Johannes Wöstemeyer, Jürgen Voigt

**Affiliations:** a Charité – University Medicine Berlin, Institute of Biochemistry, D-10117 Berlin, Germany; b Friedrich-Schiller-University Jena, Chair of General Microbiology and Microbial Genetics, D-07743 Jena, Germany

**Keywords:** Aspartic protease, Cleavage sites, Cocoa, *In-vitro* proteolysis, Mass spectrometry, Peptides

## Abstract

The data provide information in support of the research article, “The cleavage specificity of the aspartic protease of cocoa beans involved in the generation of the cocoa-specific aroma precursors” (Janek et al., 2016) [1]. Three different protein substrates were partially digested with the aspartic protease isolated from cocoa beans and commercial pepsin, respectively. The obtained peptide fragments were analyzed by matrix-assisted laser-desorption/ionization time-of-flight mass spectrometry (MALDI-TOF/TOF-MS/MS) and identified using the MASCOT server. The N- and C-terminal ends of the peptide fragments were used to identify the corresponding *in-vitro* cleavage sites by comparison with the amino acid sequences of the substrate proteins. The same procedure was applied to identify the cleavage sites used by the cocoa aspartic protease during cocoa fermentation starting from the published amino acid sequences of oligopeptides isolated from fermented cocoa beans.

**Specifications Table**

**Table:** 

Subject area	*Biochemistry*
More specific subject area	*Protease cleavage specificity*
Type of data	*Tables*
How data was acquired	*Peptide mixtures obtained by cleavage of different substrate proteins with purified cocoa aspartic protease or pepsin were analyzed by liquid chromatography-MALDI-TOF/TOF-MS/MS using a 4700 proteomics Analyzer (Applied Biosystems, Framingham,MS) of-line coupled with a Ultimate HPLC system and Probot fractionation devise (both Dionex/Thermo, Idstein, Germany). Amino acid sequences of oligopeptides isolated from fermented cocoa beans were taken from the literature.*
Data format	*Analyzed*
Experimental factors	*Samples were prepared by partial digestion of different substrate proteins with purified cocoa aspartic protease or pepsin. Prior to LC-MALDI-MS/MS analyses, the peptide mixtures were modified by reduction and alkylation of cysteine residues with dithiotreitol and iodoacetamide.*
Experimental features	*Generation of oligopeptide mixtures by digestion of substrate proteins with purified cocoa aspartic protease or pepsin, fractionation and sequencing of the peptides by LC-MALDI-TOF/TOF-MS/MS and subsequent identification of the cleavage sites. Data were compared with the cleavage sites predicted from the sequences of oligopeptides isolated from fermented cocoa beans and analyzed by liquid chromatography-tandem mass spectrometry. The abundance of the different amino acid residues in the P4-P4’ positions around the cleavage sites were analyzed to get an insight into the particular cleavage specificity of the cocoa aspartic protease.*
Data source location	*Berlin, Germany, and Jena, Germany*
Data accessibility	*Data are within this article.*

**Value of the data**

1. These data characterize the cleavage sites of the cocoa aspartic protease.
2. Characterization of the cleavage specificity of an endoprotease requires the comparative analysis of the amino acid sequences around many of its cleavage sites.
3. We provide a strategy enabling the discrimination between specific and unspecific cleavage sites of an endoprotease.
4. Our data demonstrate the limitation of the identification of protease cleavage sites by LC-MALDI-TOF/TOF-MS/MS *versus* ESI-MS/MS.
5. These data will contribute to our knowledge concerning the formation of the cocoa-specific aroma precursors.

## 1 Data

Three tables are presented. Table 1 contains the cleavage sites in different substrate proteins used by the cocoa aspartic protease and pepsin, respectively, identified by *in-vitro* proteolysis. Table 2 shows the putative cleavage sites of the cocoa aspartic protease used during commercial cocoa fermentation. Table 3 shows the abundance of the different amino acids in the P4 to P4′ positions around the cleavage sites used by the cocoa aspartic protease during *in-vitro* proteolysis and cocoa fermentation, respectively.

## 2 Experimental design, materials and methods

### 2.1 Determination of cleavage sites by *in-vitro* proteolysis

Cocoa protease, the cocoa 21-kDa seed protein, and the cocoa vicilin-class(7S) globular storage protein were isolated from the acetone-dry powder of unfermented cocoa beans essentially as previously described [1], [2]. 10 mg of horse myoglobin or of the individual cocoa seed proteins in 1 ml of 20 mM sodium acetate (pH 5.0) were partially digested with either 100 µg of purified cocoa aspartic protease or 50 µg of commercial porcine pepsin (Sigma-Aldrich Chemie, Taufkirchen, Germany). The obtained peptides were modified by reduction with dithiotreitol and subsequent alkylation of the cysteine residues with iodoacetamide before being analyzed by mass spectrometry.

Liquid chromatography-MALDI-TOF/TOF-MS/MS analyses were performed on a 4700 proteomics Analyzer (ABSCIEX, Framingham, MS) off-line coupled with an Ultimate HPLC system and Probot fractionation device (both Dionex/Thermo, Idstein, Germany). LC separations were performed on an analytical column (PepMap C18, 3 μm, 150 mm×75 μm; Dionex) at a flow rate of 200 nl/min. Mobile phase (A) was 2:98 (v/v) acetonitrile/water containing 0.05% (v/v) TFA and (B) was 80:20 (v/v) acetonitrile/water containing 0.045% (v/v) TFA. Gradients were 0–10% B in 4 min, 10-50% B in 30 min, 50–100% B in 2 min. Column effluent was continuously mixed with MALDI matrix (5 mg/ml α-cyano-4-hydroxycinnamic acid in 70:30 (v/v) acetonitrile/water containing 0.1% (v/v) TFA, 1 μl/min) and spotted at 10-s intervals on 26×12 spot arrays on MALDI steel targets (Applied Biosystems, Darmstadt, Germany).

Mass spectra were acquired in a data-dependent mode. The MS spectra were recorded in the mass range of *m*/*z* 800–4000 and with the accumulation of 2000 subspectra. MS/MS spectra were measured from the five most intensive precursor ions (*S*/*N*>30). 5000–10,000 laser shots were accumulated. MS and MS/MS peak lists were generated by the “Peak to Mascot” tool of the 4000er Series Explorer v3.6. For MS/MS data analysis, MASCOT server (version 2.3, Matrixscience, London, UK) was used. Data base searches were performed using SwissProt (2015_03; 547964 protein sequences) and the following parameters: no enzyme, one missed cleavage, variable modifications: carbamidomethylation (C), oxidation (M), pyro-glu (Q), mass tolerances for MS and MS/MS: 100 ppm and 0.3 Da. Enzymatic peptides of horse myoglobin (SwissProt no. P68082), cocoa vicilin-class(7S) globulin (TrEMBL no. A0A061EM85), and the cocoa 21-kDa seed protein (SwissProt no. P32765) were accepted as identified if their MS/MS spectra provided a MASCOT score for identity with *p*<0.05.

The different cleavage sites were determined by localization of the N- and C-terminal ends of the oligopeptides within the amino acid sequence of the corresponding substrate proteins. The octapeptide sequences around the cleavage sites and their positions in the corresponding substrate proteins are listed in Table 1. Three classes of cleavage sites were found and separately listed (Table 1):

(1) Those which were exclusively cleaved by the cocoa aspartic protease (=specific cleavage sites of the cocoa enzyme),
(2) those which were cleaved both by the cocoa aspartic protease and pepsin (=unspecific cleavage sites of the cocoa enzyme) and
(3) those which were exclusively cleaved by pepsin.

### 2.2 Determination of putative in-situ cleavage sites used during cocoa fermentation

Oligopeptides isolated from fermented cocoa beans and sequenced by ESI-MS/MS mass spectrometric analyses were taken from the literature [3], [4] and used to identify the putative *in-situ* cleavage sites of the cocoa aspartic protease in the 21-kDa cocoa seed protein and in the vicilin-class(7S) globulin of the cocoa beans, respectively. The octapeptide sequences around the putative cleavage sites used in the formation of the oligopeptides isolated from fermented cocoa beans and their positions in the amino acid sequences of the 21-kDa cocoa seed protein and the cocoa vicilin-class(7S) globulin, respectively, are listed in Table 2. Since the oligopeptides generated during fermentation of the cocoa beans are more or less modified at their C-terminal ends due to the activity of a carboxypeptidase [5], prediction of the C-terminal cleavage sites is less reliable than the cleavage sites predicted from the N-terminal ends. Due to the known cleavage specificity of this particular carboxypeptidase [6], however, the putative cleavage sites corresponding to the C-terminal ends of the original cleavage products generated by the cocoa aspartic protease can be predicted with at least some reliability. When the predicted C-terminal cleavage site was assumed to be downstream from the C-terminal end of the isolated peptide, this was marked by “+CP”. Up to now, 87 different oligopeptides have been isolated from fermented cocoa beans and sequenced by mass spectrometry [3], [4]. All these oligopeptides were derived from the 21-kDa seed protein and the cocoa vicilin-class(7S) globulin, respectively [3], [4].

From the N- and C-terminal ends of these 87 oligopeptides, 98 putative cleavage sites of the cocoa aspartic protease have been predicted (Table 2), 23 of which being identical to cleavage sites detected by *in-vitro* proteolysis (Table 1, Table 2).

To get an insight into the cleavage specificity of the cocoa aspartic protease, the relative abundance of the different amino acid residues in the P4–P4′ positions around the cleavage sites have been determined (Table 3). This was done both for the cleavage sites putatively used *in-situ* (during the fermentation process) and for the cleavage sites determined by *in-vitro* proteolysis (Table 3). In the latter case, all the cleavage sites of the cocoa aspartic protease have been considered, *i.e.* without discrimination between specific and unspecific cleavage sites as done in Table 1. Considerable differences have been observed for the relative abundance of some amino acids in the P4–P4′ positions between the *in-situ* (used during fermentation) and the *in-vitro* cleavage sites, respectively (Table 3). Analysis of chemical compounds by MALDI-TOF-MS used for the identification of peptide fragments generated during *in-vitro* proteolysis [1] is restricted to ions with *m*/*z*>799, due to ions generated from the matrix components. As recently reported, most peptides present in fermented cocoa beans, however, have molecular masses below this limit [3], [4]. Therefore, considerably more peptides and their corresponding N- and C-terminal ends can be detected and analyzed by LC-ESI-MS/MS than by LC-MALDI-TOF/TOF-MS/MS.

## Figures and Tables

**Table 1:** Specific and common cleavage sites of cocoa aspartic protease and pepsin in different protein substrates^a^.

**Substrate**	**Cleavage sites specific for the cocoa protease**		**Common cleavage sites of cocoa protease and pepsin**		**Cleavage sites specific for pepsin**	
	**P4–P4′**	**Position**	**P4–P4′**	**Position**	**P4–P4′**	**Position**
**Myoglobin** (SwissProt no. P68082)	EWQQ|VLNV	7–14	DGEW|QQVL	5–12	GEWQ|QVLN	6–13
	WQQV|LNVW	8–15	QQVL|NVWG	9–16	VLNV|WGKV	11–18
	FDKF|KHLK	44–51	LNVW|GKVE	12–19	NVWG|KVEA	13–20
	LKTE|AEMK	50–57	HGQE|VLIR	25–32	GKVE|ADIA	16–23
	EDLK|KHGT	60–67	GQEV|LIRL	26–33	KVEA|DIAG	17–24
	AIIH|VLHS	111–118	QEVL|IRLF	27–34	VEAD|IAGH	18–25
	IHVL|HSKH	113–120	LIRL|FTGH	30–37	AGHG|QEVL	23–30
	HVLH|SKHP	114–121	TVVL|TALG	67–74	GHGQ|EVLI	24–31
	VLHS|KHPG	115–122	PIKY|LEFI	101–108	EVLI|RLFT	28–35
	HPGD|FGAD	120–127	KYLE|FISD	103–110	PETL|EKFD	38–45
	FRND|IAAK	139–146	YLEF|ISDA	104–111	HLKT|EAEM	49–56
	AKYK|ELGF	145–152	FISD|AIIH	107–114	KTEA|EMKA	51–58
	YKEL|GFQG	147–154	ISDA|IIHV	108–115	EAEM|KASE	53–60
			MTKA|LELF	132–139	GGIL|KKKG	74–81
			ALEL|FRND	135–142	EAEL|KPLA	84–91
			KYKE|LGFQ	146–153	PGDF|GADA	121–128
					QGAM|TKAL	129–136
					GAMT|KALE	130–137
					TKAL|ELFR	133–140
					KALE|LFRN	134–141
					LELF|RNDI	136–143
					AAKY|KELG	144–151
					ELGF|QD^−−^	149–154

**Cocoa 21-kDa seed protein** (SwissProt no. P32765)	GGLA|LGRA	57–64	VANA|ANSP	23–30	GRAT|GQSC	62–69
	GLAL|GRAT	58–65	YYVL|SSIS	45–52	CPEI|VVQR	69–76
	ATGQ|SCPE	64–71	EIVV|QRRS	71–78	VRVS|TDVN	98–105
	GKWW|VTTD	132–139	IVVQ|RRSD	72–79	NIEF|VPIR	105–112
	GYKF|RFCP	163–170	PVIF|SNAD	85–92	PIRD|RLCS	110–117
	KFRF|CPSV	165–172	VIFS|NADS	86–93	TSTV|WRLD	118–125
			AGKW|WVTT	131–138	AGVL|GYKF	159–166
			PNTL|CSWF	147–154	SVCD|SCTT	171–178
			TLCS|WFKI	149–156	SDDD|GQIR	187–194
			LCSW|FKIE	150–157	IRLA|LSDN	193–200
			CSWF|KIEK	151–158	RLAL|SDNE	194–201
			QIRL|ALSD	192–199		
			ASKT|IKQV	209–216		

**Cocoa vicilin** (TrEMBL no. A0A061EM85)	NDYR|LAMF	50–57	PKRR|SFQT	17–24	RSEE|EEGQ	1–8
	ENKE|SYNV	91–98	RRSF|QTRF	19–26	PYYF|PKRR	13–20
	TVYV|VSQD	111–118	EGNF|KILQ	30–37	YYFP|KRRS	14–21
	GMFR|KAKP	190–197	FKIL|QRFA	33–40	YFPK|RRSF	15–22
	KAKP|EQIR	194–201	LQRF|AENS	36–43	RSFQ|TRFR	20–27
	AKPE|QIRA	195–202	KGIN|DYRL	47–54	FQTR|FRDE	22–29
	KPEQ|IRAI	196–203	GIND|YRLA	48–55	QTRF|RDEE	23–30
	ERLA|INLL	216–223	DYRL|AMFE	51–58	KILQ|RFAE	34–41
	FKLN|QGAI	257–264	RLAM|FEAN	53–60	ILQR|FAEN	35–42
	VPHY|NSKA	266–273	CDAE|AIYF	70–77	NPNT|FILP	60–67
	GYAQ|MACP	284–291	EAIY|FVTN	73–80	DAEA|IYFV	71–78
	VTFF|ASKD	343–350	TITF|VTHE	84–91	AEAI|YFVT	72–79
	LVDN|IFNN	395–402	TVVS|VPAG	102–109	AIYF|VTNG	74–81
			SVPA|GSTV	105–112	GTIT|FVTH	83–90
			STVY|VVSQ	110–117	VTHE|NKES	88–95
			TIAV|LALP	124–131	KESY|NVQR	93–100
			VLAL|PVNS	127–134	ESYN|VQRG	94–101
			KYEL|FFPA	137–144	YNVQ|RGTV	96–103
			ELFF|PAGN	139–146	VQRG|TVVS	98–105
			NKPE|SYYG	147–154	RGTV|VSVP	100–107
			YGAF|SYEV	153–160	GTVV|SVPA	101–108
			YEVL|ETVF	158–165	VVSV|PAGS	103–110
			REKL|EEIL	169–176	AGST|VYVV	108–115
			KLEE|ILEE	171–178	GSTV|YVVS	109–116
			EEIL|EEQR	173–180	LTIA|VLAL	123–130
			QIRA|ISQQ	199–206	IAVL|ALPV	125–132
			GERL|AINL	215–222	PGKY|ELFF	135–142
			AINL|LSQS	219–226	GKYE|LFFP	136–143
			NGRF|FEAC	233–240	YELF|FPAG	138–145
			AVSA|FKLN	253–260	PESY|YGAF	149–156
			NQGA|IFVP	260–267	YYGA|FSYE	152–159
			KATF|VVFV	272–279	GAFS|YEVL	154–161
			SGRQ|DRRE	302–309	AFSY|EVLE	155–162
			GRQD|RREQ	303–310	FSYE|VLET	156–163
			RQDR|REQE	304–311	EVLE|TVFN	159–166
			EETF|GEFQ	316–323	ETVF|NTQR	162–169
			TFGE|FQQV	318–325	QQGM|FRKA	188–195
			FGEF|QQVK	319–326	QGMF|RKAK	189–196
			GDVF|VAPA	332–339	LAIN|LLSQ	218–225
			AVTF|FASK	342–349	INLL|SQSP	220–227
			AVAF|GLNA	355–362	GRFF|EACP	234–241
			QRIF|LAGK	366–373	FSQF|QNMD	244–251
			KKNL|VRQM	373–380	VSAF|KLNQ	254–261
			EAKE|LSFG	383–390	AFKL|NQGA	256–263
			FSKL|VDNI	392–399	GAIF|VPHY	262–269
			ESYF|MSFS	405–412	FVVF|VTDG	275–282
					CPHL|SRQS	290–297
					SRQS|QGSQ	294–301
					RQSQ|GSQS	295–302
					SQGS|QSGR	297–304
					QGSQ|SGRQ	298–305
					GSQS|GRQD	299–306
					SQSG|RQDR	300–307
					EEET|FGEF	315–322
					PGDV|FVAP	331–338
					PLNA|VAFG	352–359
					NAVA|FGLN	354–361
					AFGL|NAQN	357–364
					FGLN|AQNN	358–365
					NNQR|IFLA	364–371
					RIFL|AGKK	367–374
					IFLA|GKKN	368–375
					FLAG|KKNL	369–376
					VRQM|DSEA	377–384
					RQMD|SEAK	378–385
					QMDS|EAKE	379–386
					MDSE|AKEL	380–387
					GVPS|KLVD	390–397
					DNIF|NNPD	397–404
					NNPD|ESYF	401–408
					PDES|YFMS	403–410
					SQQR|QRGD	412–419
					QQRQ|RGDE	413–420

a Octapeptide sequences around the cleavage sites for the cocoa aspartic protease and pepsin, respectively, detected by partial proteolysis of myoglobin, the cocoa 21-kDa seed protein, and the cocoa vicilin-class(7S) globulin. Data were separately listed for sites exclusively cleaved by the cocoa aspartic protease and pepsin, respectively, and those cleaved by both proteases (=unspecific cleavage sites).

**Table 2:** Putative cleavage sites of the cocoa aspartic protease predicted from oligopeptides isolated from fermented cocoa beans.

**Substrate**	**Putative cleavage site**^a^	**Position**^b^	**N- or C-terminal localization of the cleavage site**^c^	**Cleavage site also detected*****in vitro*****[1]**	**References**
**Cocoa 21-kDa seed protein** (SwissProt no. P32765)	VANA|ANSP	23–30	N-terminal	yes	[3]
	SPVL|DTDG	29–36	C-terminal	no	[3]
	YYVL|SSIS	45–52	N-terminal	yes	[3]
	SSIS|GAGG	49–56	N-terminal	no	[3]
	GGGL|ALGR	56–63	C-terminal	no	[3]
	IVVQ|RRSD	72–79	N-terminal	yes	[3]
	SDLD|NGTP	78–85	N-terminal	no	[3]
	PVIF|SNAD	85–92	N- and C-terminal	no	[3]
	FSNA|DSKD	88–95	N-terminal	no	[3]
	DVVR|VSTD	96–103	N-terminal	no	[3]
	TDVN|IEFV	102–109	N- and C-terminal	no	[3]
	NIEF|VPIR	105–112	C-terminal	no	[3]
	CSTS|TVWR	116–123	N-terminal	no	[3]
	STVW|RLDN	119–126	N-terminal	no	[3]
	WRLD|NYDN	122–129	C-terminal	no	[3]
	LALS|DNEW	195–202	N-terminal	no	[3]
	AWMF|KKAS	203–210	C-terminal	no	[3]
					
**Cocoa vicilin** (TrEMBL no. A0A061EM85)	EGQQ|RNNP	6–13	N- and C-terminal	no	[3], [4]
	GQQR|NNPY	7–14	N-terminal	no	[3], [4]
	QQRN|NPYY	8–15	N-terminal	no	[4]
	QRNN|PYYF	9–16	N-terminal	no	[4]
	PYYF|PKRR	13–20	C-terminal+CP	no	[4]
	YFPK|RRSF	15–22	N- and C-terminal	no	[3], [4]
	FPKR|RSFQ	16–23	N-terminal	no	[4]
	RRSF|QTRF	19–26	C-terminal	yes	[3], [4]
	RSFQ|TRFR	20–27	N-terminal	no	[3]
	TRFR|DEEG	24–31	N-terminal	no	[3]
	RDEE|GNFK	27–34	N- and C-terminal	no	[3], [4]
	EEGN|FKIL	29–36	N-terminal	no	[3]
	EGNF|KILQ	30–37	N- and C-terminal	yes	[3], [4]
	FKIL|QRFA	33–40	C-terminal	yes	[3]
	KILQ|RFAE	34–41	C-terminal	no	[4]
	SPPL|KGIN	43–50	N-terminal	no	[4]
	KGIN|DYRL	47–54	C-terminal	yes	[4]
	INDY|RLAM	49–56	N-terminal	no	[4]
	RLAM|FEAN	53–60	C-terminal+CP	yes	[4]
	NPNT|FILP	60–67	N-terminal	no	[4]
	ILPH|HCDA	65–72	C-terminal	no	[4]
	YFVT|NGKG	76–83	N-terminal	no	[3]
	VTNG|KGTI	78–85	N-terminal	no	[4]
	TITF|VTHE	84–91	C-terminal±CP	yes	[3], [4]
	THEN|KESY	89–95	N-terminal	no	[3]
	YNVQ|RGTV	96–103	N- and C-terminal	no	[3], [4]
	TVVS|VPAG	102–109	C-terminal	yes	[4]
	VLAL|PVNS	127–134	N-terminal	yes	[4]
	LPVN|SPGK	129–138	N-terminal	no	[4]
	PGKY|ELFF	135–142	C-terminal	no	[4]
	FPAG|NNKP	142–149	N-terminal	no	[3]
	AGNN|KPES	144–151	N-terminal	no	[4]
	NKPE|SYYG	147–154	C-terminal	no	[3]
	KPES|YYGA	148–155	N- and C-terminal	no	[3], [4]
	FSYE|VLET	156–163	N-terminal	no	[3]
	YEVL|ETVF	158–167	C-terminal	yes	[3]
	EVLE|TVFN	159–166	C-terminal	no	[3]
	PRHR|GGER	209–217	N-terminal	no	[4]
	ERLA|INLL	216–223	N-terminal	yes	[4]
	AINL|LSQS	219–226	C-terminal+CP	yes	[4]
	INLL|SQSP	220–227	C-terminal	no	[4]
	NLLS|QSPV	221–228	C-terminal	no	[4]
	VAVS|AFKL	252–259	N-terminal	no	[4]
	AVSA|FKLN	253–260	N-terminal	yes	[4]
	FKLN|QGAI	257–264	C-terminal+CP	yes	[4]
	KLNQ|GAIF	258–265	N- and C-terminal	no	[4]
	LNQG|AIFV	259–266	N-terminal	no	[4]
	NQGA|IFVP	260–267	N- and C-terminal	yes	[4]
	QGAI|FVPH	261–268	N-terminal	no	[4]
	GAIF|VPHY	262–269	N-terminal	no	[4]
	VPHY|NSKA	266–273	C-terminal+CP	yes	[4]
	PHYN|SKAT	267–274	C-terminal	no	[4]
	HYNS|KATF	268–275	C-terminal	no	[4]
	KATF|VVFV	272–279	C-terminal+CP	yes	[4]
	SQSG|RQDR	300–307	N-terminal	no	[3]
	EQEE|ESEE	309–316	C-terminal	no	[3]
	GEFQ|QVKA	320–327	N-terminal	no	[4]
	QQVK|APLS	323–330	N-terminal	no	[3]
	KAPL|SPGD	326–333	N- and C-terminal	no	[3], [4]
	APLS|PGDV	327–334	N-terminal	no	[3]
	PLSP|GDVF	328–335	N-terminal	no	[3]
	GDVF|VAPA	332–339	N- and C-terminal	yes	[3], [4]
	VFVA|PAGH	334–341	N-terminal	no	[3]
	APAG|HAVT	337–344	N-terminal	no	[4]
	AVTF|FASK	342–349	C-terminal	yes	[3], [4]
	VTFF|ASKD	343–350	N- and C-terminal	yes	[3], [4]
	FFAS|KDQP	345–352	N-terminal	no	[3]
	FASK|DQPL	346–353	N-terminal	no	[4]
	AVAF|GLNA	355–362	C-terminal+CP	yes	[3], [4]
	LNAQ|NNQR	360–367	N-terminal	no	[4]
	NAQN|NQRI	361–368	N-terminal	no	[4]
	AQNN|QRIF	362–369	N-terminal	no	[4]
	QNNQ|RIFL	363–370	N-terminal	no	[4]
	QRIF|LAGK	366–373	C-terminal	no	[4]
	GKKN|LVRQ	372–379	N-terminal	no	[4]
	NLVR|QMDS	375–382	C-terminal	no	[4]
	AKEL|SFGV	384–391	N-terminal	no	[4]
	KELS|FGVP	385–392	N-terminal	no	[4]
	PSKL|VDNI	392–399	C-terminal+CP	no	[4]
	NPDE|SYFM	402–409	N-terminal	no	[4]
	ESYF|MSFS	405–412	C-terminal	no	[4]

a Octapeptide sequence (P4–P4′) around the putative cleavage site.

b Position of the octapeptide in the amino acid sequence of the degraded seed protein.

c Localization of the cleavage site at the N-terminal or C-terminal end of the oligopeptide, from which the cleavage site was predicted. Since the peptides formed during cocoa fermentation are modified by a carboxypeptidase [2], [5], the N-terminal cleavage sites are more reliable than the C-terminal ones. In case of the C-terminal ends of the corresponding oligopeptide, a downstream localized cleavage site was predicted, whenever the resulting peptide fragment could be modified by the cocoa carboxypeptidase [6] to the finally detected oligopeptide (indicated by “+CP”).

**Table 3:** Abundance of different amino acid residues in the P4 to P4′ positions of the predicted and experimentally detected cleavage sites of the cocoa aspartic protease.

	**P4**^a^		**P3**^a^		**P2**^a^		**P1**^a^	
	***In-situ***^b^^,^^d^	***In-vitro***^c^^,^^d^	***In-situ***^b^^,^^d^	***In-vitro***^c^^,^^d^	***In-situ***^b^^,^^d^	***In-vitro***^c^^,^^d^	***In-situ***^b^^,^^d^	***In-vitro***^c^^,^^d^
**W**	1.02	0.93	1.02	0.93	0.00	1.88	1.02	4.67
**F**	**8.16**	**6.54**	4.08	1.88	4.08	3.76	**15.30**	**20.56**
**Y**	5.10	4.67	3.06	**6.54**	4.08	3.76	3.06	3.76
**L**	4.08	5.61	**7.14**	**8.41**	**11.32**	5.54	**12.24**	**20.56**
**I**	4.08	2.80	4.08	**11.32**	**6.12**	**6.54**	1.02	0.00
**M**	0.00	0.93	0.00	0.93	1.02	0.00	1.02	0.93
**V**	**7.14**	5.61	**8.16**	**10.29**	**16.32**	**11.32**	0.00	4.67
**A**	**10.20**	**10.28**	**8.16**	4.67	**8.16**	**7.48**	**6.12**	**8.49**
**G**	**6.12**	**10.28**	**7.14**	**9.34**	3.06	3.76	5.10	0.00
**C**	1.02	1.88	0.00	0.93	0.00	0.93	0.00	0.00
**T**	5.10	**6.54**	3.06	3.76	4.08	5.54	2.04	0.93
**S**	**6.12**	2.80	**7.14**	4.67	5.10	3.76	**11.22**	3.76
**Q**	**6.12**	4.67	**7.14**	**5.54**	4.08	3.76	**9.18**	5.54
**N**	**8.16**	3.76	**6.12**	2.80	**12.24**	4.67	**13.26**	2.80
**E**	**7.14**	**11.20**	4.08	5.54	**6.12**	**8.49**	**6.12**	**10.28**
**D**	1.02	1.88	4.08	4.67	2.04	2.80	2.04	4.67
**H**	1.02	2.80	2.04	0.93	2.04	1.88	1.02	1.88
**R**	4.08	3.74	**7.14**	4.67	1.02	**9.34**	**6.12**	3.76
**K**	**7.14**	**9.34**	5.10	**9.34**	4.08	**11.32**	3.06	1.88
**P**	**7.14**	3.74	**11.22**	2.80	5.10	2.80	1.02	0.93

	**P1**′^a^		**P2**′^a^		**P3**′^a^		**P4**′^a^	
	***In-situ***^b^^,^^d^	***In-vitro***^c^^,^^d^	***In-situ***^b^^,^^d^	***In-vitro***^c^^,^^d^	***In-situ***^b^^,^^d^	***In-vitro***^c^^,^^d^	***In-situ***^b^^,^^d^	***In-vitro***^c^^,^^d^

**W**	0.00	1.88	0.00	0.00	1.02	1.88	1.02	0.93
**F**	**7.14**	**11.22**	4.08	**6.54**	**12.24**	**7.48**	**9.18**	**7.48**
**Y**	1.02	0.93	**6.12**	3.76	3.06	1.88	4.08	0.00
**L**	3.06	**10.28**	**6.12**	**6.54**	5.10	**8.41**	**6.12**	5.66
**I**	3.06	**10.28**	4.08	**8.41**	**6.12**	5.66	4.08	4.67
**M**	1.02	1.88	1.02	0.93	0.00	1.88	2.04	0.93
**V**	**9.18**	**11.21**	**7.14**	5.66	5.10	5.66	**7.14**	**8.41**
**A**	**6.12**	**8.41**	**8.16**	**10.28**	**8.16**	**8.41**	**7.14**	**8.41**
**G**	**6.12**	5.66	**9.18**	3.76	**8.16**	4.67	**6.12**	**6.54**
**C**	0.00	1.88	1.02	0.93	0.00	1.88	0.00	0.93
**T**	3.06	0.93	4.08	4.67	5.10	3.76	3.06	3.76
**S**	**9.18**	**7.48**	**10.20**	**11.21**	**6.12**	**6.54**	**8.16**	**7.48**
**Q**	**7.14**	**6.54**	4.08	3.76	3.06	**9.34**	3.06	5.66
**N**	**9.18**	2.80	**9.18**	3.76	4.08	**8.41**	**6.12**	4.67
**E**	**3**.06	4.67	4.08	**9.34**	**6.12**	3.76	3.06	**7.48**
**D**	**6.12**	1.88	3.06	0.93	**7.14**	1.88	**6.12**	**8.41**
**H**	2.04	0.93	0.00	2.80	2.04	3.76	2.04	2.80
**R**	**10.20**	3.74	5.10	**10.28**	5.10	**6.54**	**8.16**	2.80
**K**	**8.16**	5.66	5.10	4.67	**7.14**	4.67	4.08	**6.54**
**P**	5.10	1.88	**8.16**	1.88	5.10	3.76	**9.18**	**6.54**

a Amino acid positions around the cleavage sites.

b Predicted from the N-terminal and C-terminal ends of oligopeptides isolated from fermented cocoa beans [3], [4].

c Detected by *in vitro* digestion of three different protein substrates with the cocoa aspartic protease (compare Table 1).

d Values are expressed in percent of all amino acids found in these positions. Values above 6% are marked in bold.
