# Supplementary material for: Dataset of cocoa aspartic protease cleavage sites
Source: Data Brief. 2016 Jun 24;8:700–8. doi: 10.1016/j.dib.2016.06.021 (PMC4950170; doi:10.1016/j.dib.2016.06.021)
Supplement: Supplementary file 1 — Supplementary material [file mmc1.doc]

All the authors declare that there is no conflict of interest.
